# The Role of Anesthesia in Sedation and Weaning From Mechanical Ventilation: A Systematic Review

**DOI:** 10.7759/cureus.82074

**Published:** 2025-04-11

**Authors:** Majed M Madkhali, Manssour M Alfaifi, Abdulrahman Y Safhi, Yasser M Shmakhi, Arwa H Alammari, Majed M Qaysi, Amaal A Hamdi, Saleha M Ayoub, Shahad A Rajhi, Roaa G Shaikhain, Abdulrahman F Alsubaie, Mohammed N Alazmi, Rafa M Hadaddi

**Affiliations:** 1 Anesthesia, Prince Mohammed bin Nasser Hospital, Jazan, SAU; 2 Department of Critical Care, King Fahad Medical City, Riyadh, SAU; 3 College of Medicine, Jazan University, Jazan, SAU; 4 Department of Emergency Medicine, Sabya General Hospital, Sabya, SAU; 5 College of Medicine, Taif University, Taif, SAU; 6 College of Medicine, Jouf University, Sakaka, SAU; 7 Department of Emergency Medicine, King Fahad Central Hospital, Jazan, SAU

**Keywords:** anesthesia, critical care, dexmedetomidine, intensive care, mechanical ventilation, methadone, patient outcomes, sedation, sequential sedation, weaning

## Abstract

Mechanical ventilation is a critical component of care in ICUs, yet its prolonged use can result in significant complications. Effective sedation strategies play a pivotal role in facilitating the discontinuation of mechanical ventilation and minimizing associated adverse outcomes. This systematic review evaluates the impact of anesthetic-based sedation methods on optimizing the process of weaning adult patients from mechanical ventilation in intensive care settings. A comprehensive literature search was conducted across major databases, including PubMed, Web of Science, Scopus, the Virtual Health Library, and Cochrane CENTRAL, up to March 10, 2024, following established systematic review guidelines. Eligible studies included randomized controlled trials and observational research comparing anesthetic agents with conventional sedation techniques, with outcomes such as weaning duration, extubation success, length of stay in the ICU, incidence of delirium, sedation quality, adverse events, and mortality. Study quality was assessed using a validated methodological checklist. Out of 1,649 records screened, five studies met the inclusion criteria. Results indicated that dexmedetomidine was associated with shorter weaning times and reduced anxiety, agitation, and delirium compared to traditional sedation. Sequential sedation protocols, particularly transitions from midazolam to dexmedetomidine, yielded improved clinical outcomes, while enteral methadone significantly reduced weaning duration compared to fentanyl. Despite higher daily costs, anesthetic agents demonstrated favorable economic outcomes due to shorter intensive care stays. These findings suggest that targeted anesthetic sedation strategies may enhance the weaning process and improve overall patient outcomes, underscoring the need for further large-scale studies to validate and standardize these approaches.

## Introduction and background

Mechanical ventilation is a critical supportive therapy widely utilized in ICUs to manage patients with respiratory failure. Despite its lifesaving potential, prolonged mechanical ventilation can lead to significant complications, including ventilator-associated pneumonia, diaphragmatic dysfunction, and increased mortality rates [1,2]. Therefore, strategies aimed at safely minimizing the duration of mechanical ventilation have become essential aspects of intensive care practice.

Sedation plays a pivotal role in the management of mechanically ventilated patients by reducing anxiety, agitation, and patient-ventilator asynchrony, thus facilitating effective ventilation [3,4]. However, excessive sedation is associated with numerous adverse outcomes, such as prolonged mechanical ventilation, delayed weaning, increased length of stay in the ICU, and a higher incidence of delirium [5,6]. Consequently, optimizing sedation regimens remains an important clinical goal.

Traditionally, benzodiazepines, particularly midazolam, have been the cornerstone of sedation practice in ICUs due to their anxiolytic, amnesic, and hypnotic properties [7]. However, accumulating evidence indicates that benzodiazepines may prolong sedation and mechanical ventilation due to their active metabolites and potential accumulation, especially with continuous infusion [8,9].

Recently, alternative sedative agents such as propofol, dexmedetomidine, and ketamine have emerged as potential candidates due to their favorable pharmacokinetic profiles and reduced delirium risk compared to benzodiazepines [10,11]. Propofol offers rapid onset and clearance, allowing precise sedation control and potentially faster weaning from mechanical ventilation; however, it carries risks of hypotension, hypertriglyceridemia, and rare but serious propofol infusion syndrome [3,12].

Dexmedetomidine, an alpha-2 adrenergic agonist, has gained particular attention due to its ability to provide sedation with minimal respiratory depression, preserving patient cooperation and spontaneous breathing during weaning trials [13]. Studies suggest that dexmedetomidine-based sedation might shorten the duration of mechanical ventilation and reduce the incidence of delirium, potentially leading to earlier discharge from the ICU and improved patient outcomes [10,14].

The practice of sequential sedation, where one sedative agent is transitioned to another based on clinical conditions, has been explored to combine the benefits of different sedatives while minimizing their individual drawbacks [15,16]. Evidence indicates that sequential sedation strategies could enhance sedation management, reduce sedation-related adverse effects, and shorten mechanical ventilation duration, thus improving clinical outcomes and ICU efficiency [15,17].

However, optimal sedation strategies remain controversial, with significant variability in clinical practices globally. The ideal sedation method should effectively control patient distress and facilitate weaning from mechanical ventilation while minimizing adverse effects, cognitive dysfunction, and ICU resource use [4,9]. This variability underscores the need for clear, evidence-based guidance regarding anesthetic sedation methods and their impact on ICU patient outcomes.

This systematic review aims to evaluate and synthesize the existing evidence regarding the role of anesthetic sedation strategies in facilitating weaning from mechanical ventilation in ICU patients. The review will specifically focus on comparing anesthetic agents against traditional sedation approaches, assessing their effects on key clinical outcomes such as weaning duration, extubation success, delirium incidence, length of stay in the ICU, and sedation quality. The results will provide clinicians with evidence-based insights to enhance sedation practices, optimize mechanical ventilation weaning protocols, and improve patient care quality in ICU settings.

## Review

Literature search strategy

This systematic review was registered with the International Prospective Register of Systematic Reviews (PROSPERO). We adhered to the Preferred Reporting Items for Systematic reviews and Meta-Analyses (PRISMA) guidelines for conducting and reporting this review [18]. Our search strategy covered four major online databases: PubMed, Web of Science, Scopus, Virtual Health Library, and Cochrane Central Register of Controlled Trials, from inception to March 10, 2024. Specific keywords utilized included anesthesia, intensive care unit, sedation, weaning, and mechanical ventilation. These keywords were strategically combined using Boolean operators, and the search was appropriately tailored for each database. Filters were applied to restrict results to English-language articles involving human subjects. Furthermore, reference lists of included studies were manually reviewed to identify additional relevant studies potentially missed in the initial database search.

Eligibility criteria

We set the selection criteria using the population, intervention, comparison, outcome (PICO) framework. We included randomized controlled trials and observational studies (prospective or retrospective cohort studies) published in English that involved adult ICU patients receiving mechanical ventilation under sedation, evaluated sedation using anesthetic agents such as dexmedetomidine, propofol, methadone, or midazolam, and compared anesthetic sedation methods to conventional sedation approaches, placebo, or no sedation. We also included studies that reported primary outcomes such as weaning duration and extubation success, or secondary outcomes including ICU stay length, total duration of mechanical ventilation, sedation quality, adverse events, delirium or anxiety incidence, mortality, and ICU-related costs. We excluded pediatric or neonatal studies, animal or non-human trials, case reports, case series, reviews, editorials, conference abstracts, and studies published in languages other than English.

Study selection

Two reviewers independently screened the titles and abstracts of the retrieved articles using predetermined eligibility criteria. Any disagreements or discrepancies were resolved by a third reviewer until a consensus was reached. The full text of the included articles was further analyzed, and the following data were extracted: sample size, patient diagnosis and condition, sedation method including anesthetic agents used, dosage and duration of sedation, ICU setting, comparator type, comparator dosage and duration, primary and secondary outcomes, and main results. Any potential conflicts were resolved by a third reviewer.

Quality appraisal

The methodological quality of the included studies was independently assessed by two reviewers using the modified Downs and Black scale for clinical trials [19]. The scale consists of 27 questions rating four categories: reporting, external validity, internal validity, and power. Studies are considered of excellent quality when the final score ranges from 26 to 28, good quality if the score ranges from 20 to 25, fair quality if the score ranges from 19 to 15, and poor if the score is 14 or less. Any disagreements or discrepancies were resolved by discussion until a consensus was reached.

Study selection

**Figure 1:**
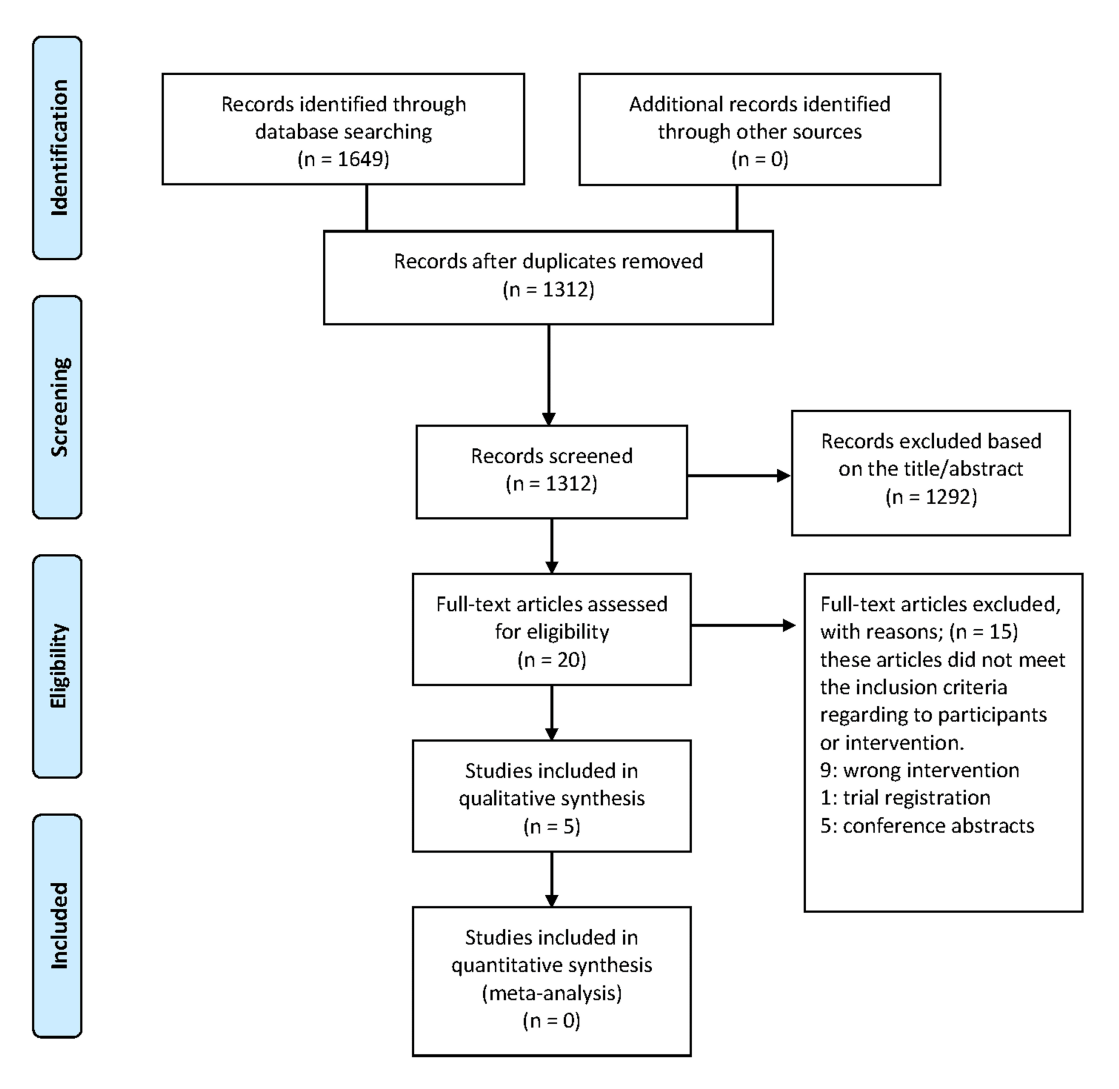
Flow diagram of the study selection process according to PRISMA guidelines PRISMA, Preferred Reporting Items for Systematic reviews and Meta-Analyses

The initial search identified a total of 1,649 records through database searches, with no additional records found through other sources, yielding a total of 1,649 records. After removing duplicates, 1,312 records remained for screening. During the title and abstract screening phase, 1,292 studies were excluded based on their relevance, leaving 20 full-text articles to be assessed for eligibility, as illustrated in the PRISMA flow diagram (Figure 1). During the full-text screening, a total of 15 articles were excluded for various reasons: eight articles had the wrong intervention, one was excluded due to being a trial registration, and five were conference abstracts, thus not meeting the inclusion criteria. Ultimately, five studies [15,16,20,21] met the eligibility criteria and were included in the qualitative synthesis. However, no studies were found eligible for quantitative synthesis (meta-analysis).

Study characteristics

Table 1 summarizes the main characteristics of five clinical studies conducted between 2010 and 2022, evaluating different sedation strategies in ICU patients undergoing mechanical ventilation. These studies span diverse geographic locations, including Sweden, Australia, Italy, Brazil, and China, reflecting varied clinical practices and patient populations. Study designs varied considerably: Zhou et al. and Conti et al. conducted prospective randomized controlled trials [15,16]; Wanzuita et al. carried out a prospective double-blind randomized controlled trial [17]; Nunes et al. conducted a retrospective analysis [20]; and Shehabi et al. executed a prospective observational study [21]. Sample sizes ranged from smaller cohorts (20 patients in Conti et al. [16]; 28 patients in Shehabi et al. [21]) to larger studies (152 patients in Nunes et al. [20]; 252 patients in Zhou et al. [15]), indicating different scales of investigation.

**Table 1:** Study characteristics and outcomes of sedation interventions in mechanically ventilated patients AI, asynchrony index; DEX, dexmedetomidine; EAdi, electrical activity of diaphragm; HRQoL, health-related quality of life; MAAS, Modified Ramsay Sedation Scale; MV, mechanical ventilation; PTSD, post-traumatic stress disorder; RASS, Richmond Agitation Sedation Scale; SBT, spontaneous breathing trial; SOC, standard of care; SOCDEX, standard of care plus dexmedetomidine

Author	Country	Study design	Sample size	Age (mean ± SD, range)	Gender (M/F)	Patient diagnosis and condition	Intervention (anesthetic type)	Intervention dosage and duration	ICU setting	Comparator type	Comparator dosage and duration	Outcomes	Results
Zhou et al. [15]	China	Randomized controlled trial	252 patients	Group M-D: 54.5 ± 14.5, Group M-P: 51.0 ± 16.0, Group M: 50.8 ± 15.4 years	M-D: 50/27, M-P: 56/22, M: 52/21	Critically ill adult patients undergoing mechanical ventilation (≥72 hours), initially receiving midazolam, needing sedation after passing SBT	Sequential midazolam to DEX (M-D)	DEX: 0.2-0.7 µg/kg/h; max 1.4 µg/kg/h. Duration: median 24 hours	Medical and surgical ICU (single center)	Midazolam alone (M) or sequential midazolam to propofol (M-P)	Midazolam: 0.04-0.20 mg/kg/h; propofol: 0.5-3.0 mg/kg/h. Median duration: M-P 26 hours, M 25 hours	Primary: MV weaning time; secondary: recovery and extubation times, delirium incidence, length of ICU/hospital stay, sedation quality	M-D had significantly shorter weaning (25.0 hours vs. 49.0 hours, p = 0.025), faster recovery/extubation, lower delirium (19.5% vs. 43.8%, p = 0.002), and more time in target sedation vs. midazolam alone. M-D superior to M-P for recovery, extubation, and sedation quality (all p < 0.001)
Conti et al. [16]	Italy	Prospective, open-label, randomized multicenter trial	20 patients	Mean 68.8 ± 15.7 (range 39-88 years)	11 M / 9 F	Difficult-to-wean patients (failed first weaning trial), mechanically ventilated patients requiring sedation (RASS score +1 to −2)	DEX	Dose: 0.2-1.4 µg/kg/h; median duration: 31.5 hours (range 18-174 hours)	General ICUs of university hospitals	Propofol	Dose: 0.3-4 mg/kg/h; median duration: 47.9 hours (range 22-113 hours)	Primary: AI; secondary: respiratory parameters, peak EAdi, time to extubation, duration of ICU stay	AI significantly lower with DEX at 12 hours (2.68% vs. 9.10%, p < 0.05). No significant differences in respiratory parameters, time to extubation (25.18 hours DEX vs. 57.33 hours Prop, p = 0.958), ICU stay (6.02 days DEX vs. 10.06 days Prop, p = 0.742), adverse events low in both groups
Wanzuita et al. [17]	Brazil	Prospective, double-blind randomized controlled trial	68 patients	Methadone: 43 ± 18 years, control: 45 ± 17 years	Methadone: 27 M/10 F, Control: 26 M/5 F	Adult critically ill patients, mechanically ventilated ≥5 days, receiving fentanyl infusion ≥5 days	Methadone (enteral)	Methadone 10 mg capsule enterally every six hours, IV fentanyl reduced by 20%/day (median weaning: five days)	Adult ICUs of four general hospitals	Fentanyl (IV)	Fentanyl infusion reduced gradually by 20%/day (median weaning: seven days)	Primary: MV weaning time; secondary: total MV duration, ICU stay, hospital stay	Methadone significantly reduced MV weaning time among successfully weaned patients (four vs. seven days, HR: 2.06; 95% CI: 1.17-3.63; p < 0.004). Higher probability of successful weaning by day 5 in the methadone group (HR: 2.64; p < 0.02). No significant difference in total MV duration, ICU/hospital stay, or mortality
Nunes et al. [20]	Sweden	Retrospective study	152	62 years (range: 19-86)	95 M / 57 F	Adult ICU patients on mechanical ventilation (≥24 hours), mostly medical (82%), predominant causes of respiratory failure/sepsis	DEX alone, midazolam and/or propofol (SOC), SOCDEX	DEX: 54.8 ± 31.3 µg/h; propofol: SOC: 102.9 ± 78.2 mg/h, SOCDEX: 76.6 ± 126 mg/h; midazolam: SOC: 1.6 ± 1.8 mg/h, SOCDEX: 19.0 ± 15.0 mg/h; median sedation durations: DEX: 5.4 hours, SOC: 17.8 hours, SOCDEX: 26.3 hours	General ICUs (15 Swedish ICUs)	SOC: midazolam and/or propofol	Propofol: 102.9 ± 78.2 mg/h; midazolam: 1.6 ± 1.8 mg/h; median weaning: 17.8 h	Primary: weaning time; secondary: MV duration, ICU length of stay, anxiety, delirium, HRQoL, PTSD, ICU costs	DEX alone had significantly shorter weaning (5.4 hours vs. 17.8 hours SOC, 26.3 hours SOCDEX, p < 0.001). Anxiety: DEX (0%), SOC (9%), SOCDEX (24%). Very low delirium rates. Better HRQoL in DEX group (p = 0 .024 vs. SOCDEX). ICU costs are higher during weaning but lower overall in the DEX group
Shehabi et al. [21]	Australia	Prospective observational study	28 patients (30 episodes)	Median 70.5 years (IQR 51-76)	96.7% M, 3.3% F	Critically ill, mechanically ventilated (≥24 hours) adults who developed agitation/delirium upon sedation withdrawal and failed conventional weaning	DEX	Infusion started at 0.4 µg/kg/h for 2 hours; median max dose: 0.7 µg/kg/h (range 0.4-1.0 µg/kg/h); median duration: 62 hours (24-252 hours)	General ICU (tertiary medical/surgical)	Conventional sedation (midazolam, propofol, haloperidol)	Midazolam (1-5 mg/h), propofol (30-300 mg/h), duration variable (up to 48 hours pre-intervention)	Primary: proportion of patients achieving target sedation level (MAAS 2-4); secondary: total ventilation time, weaning success, additional sedation requirements, adverse events	Within six hours, 93.3% achieved the target MAAS (vs. 23.3% at baseline, p < 0.001). Successful weaning/extubation was achieved in 73.3% of episodes. Median post-infusion ventilation: 70 hours (28-96 hours). DEX rapidly resolved agitation, facilitating successful ventilatory weaning after conventional therapy failure

Patient age groups were relatively consistent, generally around 60-70 years, with specific means ranging from approximately 43 years (Wanzuita et al. [17], methadone group) up to median ages around 70.5 years. Gender distribution was predominantly male across studies, particularly pronounced in Shehabi et al. [21], where 96.7% were male, whereas the other studies showed less extreme male predominance.

Patient diagnoses and conditions typically involved critically ill adults requiring prolonged mechanical ventilation, presenting challenges in sedation and weaning. The predominant conditions included respiratory failure, sepsis, difficult-to-wean states, agitation, and delirium. Specifically, Nunes et al. [20] focused primarily on medical ICU patients with respiratory failure or sepsis, whereas Conti et al. [16] targeted difficult-to-wean patients who previously failed weaning attempts.

Dexmedetomidine was the most commonly studied sedation agent, examined explicitly in four of the five studies [15,16,20,21]. Intervention dosages varied widely, typically administered within a range of 0.2-1.4 µg/kg/h. The duration of dexmedetomidine administration also varied significantly, from shorter periods (median 5.4 hours, Nunes et al. [20]) to extended infusions (median 62 hours, Shehabi et al. [21]). Wanzuita et al. [17] differed distinctly by assessing enteral methadone, administered as 10 mg capsules every six hours, gradually replacing intravenous fentanyl sedation.

Comparator strategies generally involved conventional sedation approaches, primarily midazolam or propofol, administered variably according to local ICU standards. Dosage ranges were notably diverse; midazolam was dosed from as low as 0.04 mg/kg/h to 5 mg/h, and propofol ranged between 0.3 mg/kg/h to 300 mg/h, highlighting considerable clinical variability.

ICU settings were diverse, ranging from general ICUs across multiple centers (15 ICUs in Sweden, four hospitals in Brazil) to single-center, tertiary-level medical-surgical units (Australia and China) and general university-affiliated ICUs (Italy).

Primary outcomes consistently targeted clinically relevant sedation and weaning measures, such as mechanical ventilation weaning time [15,16,20], the proportion achieving target sedation level [21], and ventilator asynchrony index [16]. Secondary outcomes included total duration of mechanical ventilation, time to extubation, ICU and hospital length of stay, incidence of delirium, anxiety, sedation quality, patient recovery, health-related quality of life, and costs associated with ICU stays.

Quality assessment

The five studies assessed using the Modified Downs and Black Checklist showed considerable strength in reporting quality, with Conti et al. [16], Wanzuita et al. [17], Zhou et al. [15], and Shehabi et al. [21] achieving exceptionally high scores (11/11 for the first three studies and 10/11 for Shehabi et al. [21]), indicating robust clarity and completeness in reporting objectives, patient characteristics, outcomes, and findings. Nunes et al. [20] scored slightly lower (10/11) due to the absence of explicit reporting of probability distributions. External validity assessments indicated strong generalizability for three studies [16,17,20] with full scores (3/3), highlighting their representativeness to the broader ICU patient population. In contrast, Shehabi et al. [21] and Zhou et al. [15] had minor limitations, scoring 2/3 due to their single-center designs, thus slightly limiting generalizability (Table 2).

**Table 2:** Quality appraisal of the included studies The methodological quality of the included studies was independently assessed by two reviewers using the modified Downs and Black scale for clinical trials [19]. This scale includes 27 questions across four categories: reporting, external validity, internal validity (bias), and power. The studies were rated as excellent (26-28 points), good (20-25 points), fair (15-19 points), or poor (≤14 points).

Study	Reporting	External validity	Internal validity – bias	Internal validity – confounding	Power	Total	Quality rating
Zhou et al. [15]	11	2	6	6	1	26	Excellent
Conti et al. [16]	11	3	6	5	0	25	Good
Wanzuita et al. [17]	11	3	6	6	1	27	Excellent
Nunes et al. [20]	10	3	5	3	0	21	Good
Shehabi et al. [21]	10	2	4	2	0	18	Fair

Internal validity in terms of bias control revealed variability, with the highest scores (6/7) seen in randomized controlled trials by Conti et al. [16], Wanzuita et al. [17], and Zhou et al. [15], reflecting strong study design, reliable outcome measurements, and appropriate statistical analysis, although these studies lost a point due to their open-label nature and lack of outcome-assessor blinding. Nunes et al. [20] scored moderately (5/7), limited primarily by its retrospective, non-randomized design, increasing potential biases. The lowest score in this domain (4/7) was seen in Shehabi et al. [21], reflecting significant limitations due to non-randomization and absence of blinding.

Regarding internal validity related to confounding control, studies demonstrated clear variability. Wanzuita et al. [17] and Zhou et al. [15] achieved the maximum score (6/6), highlighting excellent confounding control through proper randomization and methodological rigor. Conti et al. [16] scored slightly lower (5/6), indicating strong but somewhat reduced confounding control due to its open-label design. Nunes et al. [20] had moderate confounding control (3/6) due to limited adjustments typical of retrospective designs. The lowest confounding control (2/6) was again observed in Shehabi et al. [21], indicating significant limitations.

Finally, for statistical power, Wanzuita et al. [17] and Zhou et al. [15] clearly documented adequate statistical power calculations, achieving the maximum point (1/1). However, Nunes et al. [20], Shehabi et al. [21], and Conti et al. [16] scored 0, reflecting insufficient or unreported statistical power calculations, reducing confidence in their ability to detect clinically relevant differences.

Effect of intervention

Dexmedetomidine consistently demonstrated benefits over standard sedation protocols (midazolam or propofol) in reducing the weaning duration and facilitating successful extubation. Nunes et al. [20] reported significantly shorter median weaning durations in patients sedated with dexmedetomidine alone (5.4 hours) compared to standard care (17.8 hours) and combined therapy with standard care and dexmedetomidine (26.3 hours; p < 0.001). Additionally, dexmedetomidine eliminated reintubation within 24 hours, contrasting with three cases in standard care and one in the combination group. Similarly, Shehabi et al. [21] found dexmedetomidine significantly improved sedation levels, increasing the percentage of patients achieving the target sedation from 23.3% at baseline to 93.3% within six hours (p < 0.001), with 73.3% achieving successful extubation. Zhou et al. [15] further supported these findings, observing shorter weaning durations with sequential sedation (midazolam followed by dexmedetomidine, 25.0 hours) compared to midazolam alone (49.0 hours; p = 0.025). Although Conti et al. [16] found dexmedetomidine reduced median extubation time compared to propofol (25.18 vs. 57.33 hours), this result did not reach statistical significance (p = 0.958). Additionally, Wanzuita et al. [17] highlighted that replacing fentanyl with enteral methadone significantly reduced median weaning times from seven days to four days (HR: 2.06; p < 0.004), with a higher probability of successful weaning by day 5 (HR: 2.64; p < 0.02).

Sedation quality, particularly in controlling anxiety and agitation, was notably superior with dexmedetomidine. Nunes et al. [20] observed no anxiety during weaning in the dexmedetomidine group, while it was present in 9.1% (standard care) and 24.5% (combination group). Anxiety increased post-extubation across all groups, though it remained lower with dexmedetomidine. Similarly, Shehabi et al. [21] reported agitation significantly decreased from 77% to 13% within 12 hours post-dexmedetomidine initiation (p < 0.001). Conti et al. [16] noted fewer sedation overshoot episodes (RASS score < -2) in the dexmedetomidine group compared to propofol, indicating superior control. Zhou et al. [15] also highlighted dexmedetomidine’s superior efficacy, maintaining patients within targeted sedation ranges significantly more often (71.4%) compared to midazolam alone (39.2%) and midazolam-propofol (42.9%; p < 0.001).

Dexmedetomidine appeared advantageous in reducing delirium incidence. Nunes et al. [20] found low delirium rates during weaning across all groups, with very few cases at ICU discharge. Zhou et al. [15] reinforced these results, showing significantly lower delirium incidence with sequential midazolam-dexmedetomidine sedation compared to midazolam alone (19.5% vs. 43.8%; p = 0.002).

Regarding ICU length of stay, results were mixed, although trends favored dexmedetomidine. Wanzuita et al. [17] observed no significant differences in total mechanical ventilation duration or ICU/hospital stays between methadone and fentanyl groups. Conversely, Zhou et al. [15] found significantly shorter ICU stays with dexmedetomidine (14.8 days) compared to midazolam alone (17.9 days; p = 0.006). Although Conti et al. [16] also reported shorter ICU stays with dexmedetomidine (6.02 vs. 10.06 days), the difference was not statistically significant (p = 0.742).

Adverse events associated with dexmedetomidine were limited and mild across studies. Shehabi et al. [21] recorded isolated cases of self-extubation, mild hemodynamic instability, liver enzyme elevation, and a 13% lack of efficacy. Conti et al. [16] similarly observed minimal adverse effects, including bradycardia with dexmedetomidine and respiratory complications in the propofol group. Zhou et al. [15] reported low incidences of hypotension (5.2%) and bradycardia (1.3%), with no significant differences between sedation strategies.

Dexmedetomidine’s economic impact was generally favorable despite higher daily sedation costs during weaning. Nunes et al. [20] noted higher daily sedation expenses with dexmedetomidine; however, overall ICU costs were lower due to shorter stays. Conti et al. [16] corroborated these findings, reporting substantially lower ICU costs with dexmedetomidine (€20,387) compared to propofol (€29,010), primarily driven by reduced ICU stay lengths.

Mortality outcomes were similar across sedation protocols. Wanzuita et al. [17] found no significant difference in mortality between methadone and fentanyl groups. Similarly, neither Conti et al. [16] nor Nunes et al. [20] observed sedation-related mortality, and Zhou et al. [15] reported no significant differences in ICU or hospital mortality across sedation strategies.

## Conclusions

This systematic review highlights the advantages of anesthetic sedation strategies, particularly the use of dexmedetomidine, methadone, or sequential sedation protocols, in improving weaning outcomes, enhancing sedation quality, reducing delirium, and potentially lowering ICU-related costs. The adoption of these strategies in ICU sedation protocols can significantly enhance the quality of patient care and the efficiency of resource utilization, underscoring the importance of evidence-based sedation management.
